# Colonisation of Meat by *Escherichia coli* O157:H7: Investigating Bacterial Tropism with Respect to the Different Types of Skeletal Muscles, Subtypes of Myofibres, and Postmortem Time

**DOI:** 10.3389/fmicb.2017.01366

**Published:** 2017-07-25

**Authors:** Caroline Chagnot, Annie Venien, Sandra Renier, Nelly Caccia, Régine Talon, Thierry Astruc, Mickaël Desvaux

**Affiliations:** ^1^UMR454 MEDiS, INRA, Université Clermont Auvergne Clermont-Ferrand, France; ^2^INRA, UR370 Qualité des Produits Animaux Saint-Genès Champanelle, France

**Keywords:** bacterial adhesion, foodborne pathogens, extracellular matrix, meat products, food contamination

## Abstract

*Escherichia coli* O157:H7 is an enterohaemorrhagic *E. coli* (EHEC) responsible for serious diseases, especially pediatric, and of great concern for the meat industry. Meat contamination by EHEC occurs at slaughtering, especially at dehiding stage, where bacteria can be transferred from hides to carcasses. The skeletal muscle tissues comprise four major types of myofibres, which differ in their contraction velocity and metabolism. Myofibres are surrounded by the extracellular matrix (ECM). Adhesion of *E. coli* O157:H7 to meat was investigated considering well-defined types of skeletal muscle and their constituent myofibres as well as postmortem changes in muscle, using fluorescence microscopy and immunohistochemical analyses. By analysing the adhesion of *E. coli* O157:H7 to model oxidative (soleus) and glycolytic [extensor digitorum longus (EDL)] skeletal muscles, it first appeared that differential adhesion occurred at the surface of these extreme skeletal muscle types. At a cellular level, bacterial adhesion appeared to occur essentially at the ECM. Considering the different constituent myofibres of types I, IIA, IIX and IIB, no significant differences were observed for adhering bacteria. However, bacterial adhesion to the ECM was significantly influenced by postmortem structural modifications of muscle tissues. By providing information on spatial localisation of *E. coli* O157:H7 on meat, this investigation clearly demonstrated their ability to adhere to skeletal muscle, especially at the ECM, which consequently resulted in their heterogeneous distribution in meat. As discussed, these new findings should help in reassessing and mitigating the risk of contamination of meat, the food chain and ultimately human infection by EHEC.

## Introduction

In the meat industry, the significance of enterohemorrhagic *Escherichia coli* (EHEC) as a serious public health problem is undoubtedly recognised. EHEC are foodborne pathogens that produce Shiga toxins, which are responsible for serious diseases such as HUS (haemolytic uraemic syndrome) or TTP (thrombotic thrombocytopenic purpura) (Karch et al., 2005; Tarr et al., 2008; Gould et al., 2009). Worldwide, foodborne illness associated with the consumption of meat products contaminated by EHEC has been reported and *E. coli* O157:H7 is the most frequently associated serotype. Ruminants (mainly cattle) are the natural reservoir for these bacteria and food infection occurs following direct or indirect faecal contamination (Chase-Topping et al., 2008). While the muscle masses of healthy animals are sterile (Gill, 1979), bacterial contamination of meat can occur at slaughtering, mainly upon transfer to carcasses at the dehiding stage and even when good practices are strictly respected, *i.e.* excluding evisceration accident (Giaouris et al., 2014). In fact, the respect of good hygienic practices in the beef industry during slaughtering reduces contamination of carcasses, but cannot guarantee the absence of *E. coli* O157:H7 from meat (Rhoades et al., 2009). Thus, the survival of bacteria in meat could also depend upon their ability to adhere to meat surfaces.

Whatever the mammalian species, the skeletal muscular tissue is highly structured into three main organisational levels, with the muscle cells (also called fibres) packed into fascicles, themselves regrouped to form a skeletal muscle (Schiaffino and Reggiani, 2011; Frontera and Ochala, 2015). While two extreme types of skeletal muscles can be considered, namely the white glycolytic and red oxidative muscles, a range of in-between skeletal muscles are found. These different skeletal muscles contain in the four main types of muscle fibres, namely the types I, II-A, II-X, and II-B, which are present in different proportions depending on the skeletal muscle function (Pette and Staron, 1990; Chagnot et al., 2015b). The various muscle fibres differ in their contraction velocity, i.e., slow twitch (types I) or fast twitch (types II) and in metabolism, i.e., oxidative (types I and II-A) or glycolytic (types II-X and II-B). Structural differences are also observed, such as variation of fibre cross-sectional area, depending on fibre specialisation and/or muscle type (Realini et al., 2013). Additionally, muscle fibres are surrounded by an extracellular matrix (ECM), which is divided into three types depending on its location with respect to the muscular organisational levels, namely (i) the endomysium, the deepest layer in contact with the muscle fibres, (ii) the perimysium surrounding the fascicles, and (iii) the epimysium, the outermost layer at the surface of the muscle. The three types of ECM are highly similar and are mainly composed of fibrillar collagens of types I and III. They differ slightly in the proportion of the other ECM proteins such as laminin or elastin (Gillies and Lieber, 2011; Nishimura, 2015).

According to the European Community (EC) regulation specifying the hygiene rules for foodstuffs (no. 853/2004), animal slaughter and cutting of carcasses into quarters can be carried out at room temperature before the meat is stored at low temperature. Prior to consumption, meat is deliberately stored for several days in these conditions to improve its texture and flavour. During this period, the muscles undergo postmortem modifications following a succession of significant metabolic, physical, structural and biochemical transformations resulting in meat (Bendall, 1973; Greaser, 1986). For instance, intracellular water diffuses into the extracellular space, which leads to a decrease in cell size and an increase in the intercellular spaces (Offer and Knight, 1988; Guignot et al., 1993; Huff Lonergan et al., 2010), whereas the action of endogenous proteolytic enzymes leads to the fragmentation of the muscle fibres (Taylor et al., 1995; Taylor and Koohmaraie, 1998; Waardenberg et al., 2008) and to the disintegration of the collagen fibres (Bailey and Light, 1989; Nishimura, 2010). The rate of these postmortem changes depends on the animal species, and mostly on the metabolic and contractile type of the muscle considered (Ouali, 1990; Lefaucheur, 2010).

While *E. coli* O157:H7 has the ability to attach to meat (Cabedo et al., 1997; Li and McLandsborough, 1999; Rivas et al., 2006; Chen et al., 2007), it also adheres specifically and non-specifically to some ECM fibrillar proteins (Chagnot et al., 2013a) such as some collagens (Medina, 2001; Auty et al., 2005; Chagnot et al., 2013a). Initial bacterial adhesion was shown to be strongly influenced by the bacterial physiology and environmental conditions such the temperature (Chagnot et al., 2013a). Considering that the interaction of *E. coli* O157:H7 with skeletal muscle tissue remains poorly characterised, we investigated the adhesion of *E. coli* O157:H7 to skeletal muscle fibres, considering the influence of metabolic and contractile fibre types as well as the postmortem muscle changes.

## Materials and Methods

### Bacterial Strain and Growth Conditions

For the purpose of this study, the detoxified strain (*stx*^-^) of *E. coli* O157:H7 EDL933 was used (Perna et al., 2001), i.e., *E. coli* O157:H7 CM454 (Gobert et al., 2007; Chagnot et al., 2014). For direct visualisation, the strain was transformed with the vector pSaRe-Red1 expressing the fluorescent protein mRuby and carrying the erythromycine resistant gene (300 μg mL^-1^ final concentration) (Desvaux et al., 2005; Kredel et al., 2009; Chagnot et al., 2013a). Bacteria were cultured in two different growth media, i.e., (i) DMEM (Dulbecco’s modified eagle medium, Gibco) and (ii) LB (lysogeny broth) adjusted with NaOH (0.1 M) to reach pH 7 at the time of sampling (Guedon et al., 2000; Chagnot et al., 2015a). A preculture was set up from one bacterial colony grown in DMEM or LB at 39°C (bovine temperature) in an orbital shaker at low speed (70 rpm) till the stationary phase.

For the adhesion tests, the preculture was diluted 1:100 and grown as described above. Sampling was performed during the exponential growth phase at an OD_600nm_ of 0.5 (i.e., about 10^8^ CFU ml^-1^). Chloramphenicol (170 μg ml^-1^ final concentration) was added and mixed gently to prevent *de novo* protein synthesis and growth. Therefore, no growth is occurring during the time of contact of bacterial cells with muscle in the adhesion assay and only molecular determinants expressed during anterior growth conditions are involved in the bacterial adhesion to meat. Minimal mechanical treatment was used to preserve cell surface supramolecular structures potentially involved in adhesion (Chagnot et al., 2014).

### Muscle Sampling and Maturation Processing

To control slaughter conditions, postmortem kinetics and the possibility of extracting the entire muscles without any lesion, rat muscles were used as well-recognised physiological models (Pullen, 1977; Rogers and Evans, 1993; Lagord et al., 1998; Morey-Holton and Globus, 2002; Soukup et al., 2002; Schiaffino and Reggiani, 2011; Warren et al., 2014; Naili et al., 2016). Two muscle models were chosen based on their very divergent metabolic and contraction velocity features, namely the (i) soleus containing only oxidative fibres (types I and II-A), and the (ii) extensor digitorum Longus (EDL) containing essentially glycolytic fibres, i.e., II-X (24%), II-B (46%) (and, in lower proportion, fibres of types I (4%) and II-A (18%) (Chagnot et al., 2015b).

Rats from Janvier (St-Berthevin, France) were housed in the INRA animal facility until sacrifice (“Installation Expérimentale de Nutrition, Unité de Nutrition Humaine, INRA Auvergne-Rhône-Alpes, Site de Theix”; Agreement no. C63345.14). To respect animal welfare, the rats were euthanised under anaesthesia, without pain nor suffering, in strict accordance with the recommendations and with validation by the Regional Ethics Committee (“Comité d’Ethique pour l’Experimentation Animale Auvergne”; no. C2E2A-02), which takes into account the rule of the 3Rs (replacement, reduction, refinement). After an anaesthesia by isoflurane gas, Wistar male rats (5 months old, weighing about 500 g weights) were sacrificed by decapitation. Immediately after the slaughter, the lower limbs were carved and dissected under sterile conditions. From tendon to tendon, EDL and soleus muscles were sampled without lesion. Muscles were suspended in a sterile moisture chamber maintained at 20°C to prevent contamination from microorganisms and prevent muscle alteration by drying. The artificial tension of muscle imposed by the animal carcass was mimicked by using lead ballast (1.5 g) at the bottom muscle tendon. Based on previous studies (Chagnot et al., 2015a,b), two different postmortem times were considered, i.e., *t*_0__h_ (10 min after slaughtering as the minimum required for the dissection and extraction of muscles) and *t*_24__h_ (24 h after slaughtering).

### Bacteria Adhesion to Whole Muscles and Muscle Cross-Sections

For bacterial adhesion to whole muscle, EDL and soleus muscles of at least two rats (i.e., four muscles of each type in total) were incubated statically for 30 min in 15 mL bacterial solution at 25°C. Muscles were washed by dipping three times in milliQ water to remove unattached cells. Muscles were then deposited in a sterile Petri dish adapted for inverted fluorescence microscopy (ibidi). Muscles were either incubated in LB bacterial growth conditions or in DMEM bacteria growth conditions.

The surface of muscles was observed in the fluorescence mode using an inverted microscope (Olympus IMT-2) coupled to a cooled CCD camera (Olympus DP30BW) optimised for high sensitivity fluorescence work and driven by the Cell A software v3.2 (Olympus France SAS, Rungis, France). The fluorescence light source was a mercury short arc lamp (HBO103W/2, OSRAM, Augsburg, Germany). Fluorescence acquisition was fitted with a cyanine-3 cube. Images were processed with the public domain image processing and analysis program ImageJ v1.43 (Schneider et al., 2012). The pixels corresponding to bacteria were extracted by thresholding segmentation of the light grey levels and the proportion of bacteria-overed surface was calculated.

To investigate bacterial adhesion to muscle cells, muscle sections of both right and left EDL and soleus muscles of rats were used. At *t*_0__h_ and *t*_24__h_, parts of EDL and soleus muscles were positioned on a cork plate with embedding medium (Tissue-Tek) and frozen to -160°C in isopentane with liquid nitrogen (-196°C). Based on a previously described protocol (Chagnot et al., 2015a,b), serial cross-sections (10 μm thick) were obtained using a cryostat (Microm, HM 560) and collected on glass slides. The sections were stored at -20°C under vacuum until use.

Muscle cross-sections were stained with picrosirius red, which revealed the intramuscular ECM (endomysium, perimysium and epimysium) in red and muscle fibres in yellow (Liu et al., 1994). Fibre typing was performed as previously described (Chagnot et al., 2015b). Briefly, mouse monoclonal antibodies specific to MyHC isoforms BA-D5, SC-71, BFF3 (AGRO-BIO France) were used in three different serial muscle cross sections to identify slow and fast myosin heavy chains isoforms (MyHC). The myofibre response to the different antibodies enabled us to identify the subtypes I, IIA, IIB and hybrid IIX-IIB; the types MyHC-IIX corresponded to the remaining unlabelled cells. The different primary MyHC antibodies were stained with Alexa Fluor 488-labelled goat anti-mouse IgG secondary antibody (A11001, Invitrogen). The ECM proteins, laminin, surrounding the muscle fibre, were stained using anti-laminin primary polyclonal antibody (L9393 Sigma) and a cyanine Cy3-labelled secondary antibody (111-165-008, Jackson). The cross-sections were incubated with primary antibodies. After washing, both labelled secondary antibodies were incubated to reveal the primary antibody binding. Controls were performed without primary antibody to validate the results.

Observations and image acquisitions were performed using a photonic microscope (Olympus BX 61) coupled to a high resolution digital camera (Olympus DP 71) and the Cell F software. For histological analysis, picrosirius red-stained sections were observed and images were acquired in bright field mode, whereas immunohistofluorescence images were acquired in the fluorescence mode (Cyanine 3: 550/570 nm; Alexa Fluor 488: 495/519 nm). Images of immunohistochemistry processing were recorded with the program FibTypFluo in Visilog v5.4 (Noesis, France) software to create a virtual image of the different fibre types composing the muscle section (Meunier et al., 2010).

On a histological section without any staining, the perimeter of a 1.5 cm^2^ square was traced with hydrophobic gel (PAP pen for immunostaining, Sigma), to maintain the bacterial solution on the slide. Then, 500 μL of bacterial suspension was deposited with wide bore pipette tips. Muscle sections were incubated statically in a humid chamber at 25°C for 30 min. After incubation, bacterial suspension was removed by pipetting and washed by dipping two times, in milliQ water, to remove unattached cells. The experiment was repeated three times on serial sections of both muscles for each growth condition (DMEM or LB) and postmortem time (*t*_0__h_ and *t*_24__h_).

At least six fields of view (magnification X2000) were analysed on each muscle or muscle cross-section. On each field of view, two images were systematically recorded in fluorescent and in bright field mode, respectively. Fluorescent recording allowed recording the adherent bacteria. For muscle cross-section, bright filed recording showed the muscle fibres previously identified by their fibre types using immunohistofluorescence on the other serial sections. Then, images were processed with Visilog v5.4 (Noesis, France) or ImageJ v1.43 (Schneider et al., 2012) software. The pixels corresponding to bacteria were extracted by thresholding segmentation of the light grey levels and the muscle fibres image (bright field mode) and bacteria (artificial grey scale images) were superimposed. The relative area of bacteria was assessed by quantifying the number of these pixels respective to the total number of pixels corresponding to the cell. With duplicate of serial cross-sections, ten cells of the same fibre type were compared for different conditions with respect to the postmortem time and growth medium.

### Statistical Analysis

Data were analysed with XLSTAT software 2010 (Microsoft Office, Redmond, United States) using one-way analysis of variance (ANOVA) and the Student-Newman–Keuls test, or Student’s *t*-test for date comparison of only two different populations. Given the large number of detected events (at least several 100s of pixels) used to generate the percentages of surface coverage, the binomial distribution can be approximated by a normal distribution and a classical ANOVA can be used. Results are expressed as mean ± standard error of the mean (SEM). Differences were considered as significant (^∗^*p* < 0.05), very significant (^∗∗^*p* < 0.01), highly significant (^∗∗∗^*p* < 0.001), or very highly significant (^∗∗∗∗^*p* < 0.0001).

## Results

### Differential *E. coli* O157:H7 Adhesion Occurs at the Surface of Glycolytic and Oxidative Skeletal Muscle Types and Depends on Anterior Growth Conditions

Two extreme types of skeletal muscles were considered, i.e., glycolytic and oxidative muscles, in studying the adhesion ability of *E. coli* O157:H7 to the surface of skeletal muscles (fully extracted and without any lesion). As well-recognised physiological models with such distinct metabolic and contractile features, the rat EDL and soleus skeletal muscles, respectively, were used. Regarding the bacterial cells, *E. coli* O157:H7 CM454 were placed in physiological conditions where they exhibited differential attachment features, namely the induction of specific and non-specific adhesion to the ECM when grown in LB and DMEM, respectively (Chagnot et al., 2013a). These adhesion trends were typically observed *ex vivo* using bovine gastro-intestinal tract contents, and thus are likely displayed by some bacteria after shedding.

**FIGURE 1:**
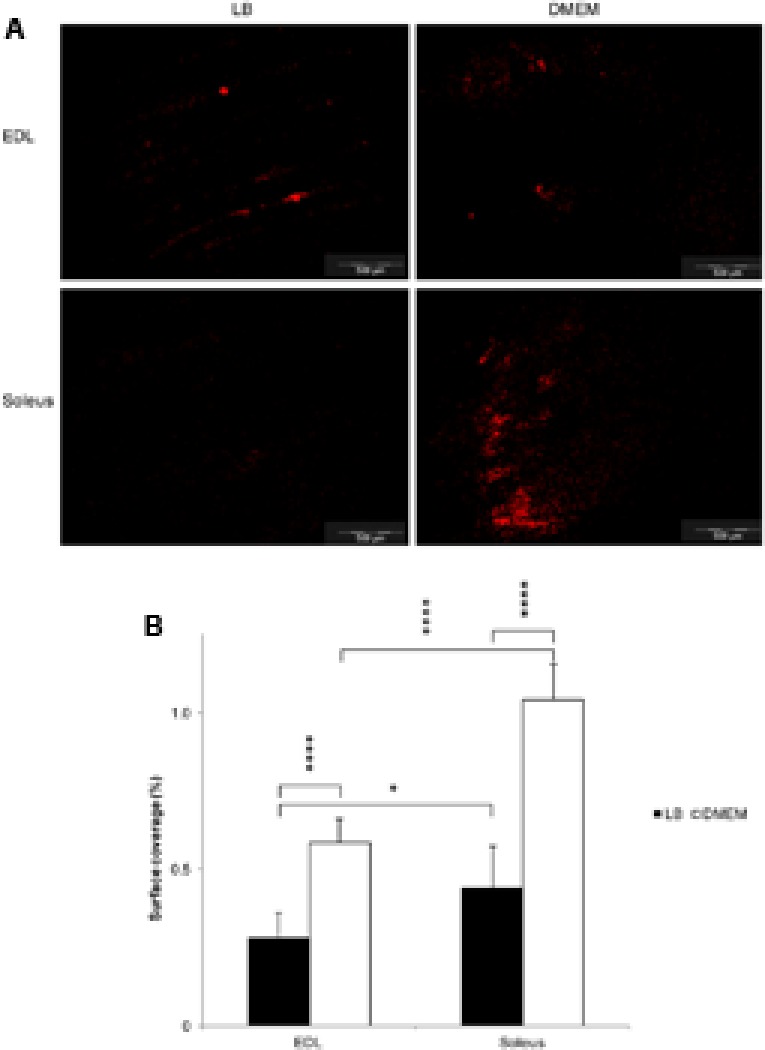
Adhesion of *Escherichia coli* O157:H7 to the surface of glycolytic and oxidative muscles as observed by epifluorescence microscopy. **(A)** Observation in epifluorescence microscopy of *E. coli* O157:H7 CM454 pSaRe-Red1 first grown in LB or DMEM and adhering to the surface of EDL (glycolytic) and soleus (oxidative) muscles at pH 7 and 25°C. Fluorescent bacteria were stained virtually in red colour. **(B)** The level of adherent bacteria assessed using fluorescence intensity was expressed as the percentage of muscle surface covered by fluorescent bacterial cells.

In LB, the surface of the muscles was covered unevenly by *E. coli* O157:H7 CM454, which appeared to adhere to specific structures of the epimysium by following the outline of the muscle fibres, which was especially apparent over the EDL (**Figure 1A**). In marked contrast, these muscles were covered all over by *E. coli* O157:H7 CM454 grown in DMEM, forming packs of bacterial cells in some areas (**Figure 1A**). As a result, bacterial surface coverage was very significantly higher at the surface of EDL or soleus when *E. coli* O157:H7 CM454 was previously grown in DMEM compared with LB (**Figure 1B**); with bacteria grown in LB, bacterial surface coverages on EDL were 0.3 and 0.4% on soleus, but reached 0.6 and 1.0% respectively, when *E. coli* O157:H7 CM454 was grown in DMEM. Besides, bacterial surface coverage appeared significantly higher at the surface of soleus than EDL whenever *E. coli* O157:H7 CM454 was grown in LB or DMEM (**Figure 1B**). Thus, it clearly appeared that some kind of tropism occurred for bacterial adhesion at the surface of these two extreme types of skeletal muscles, which could be related to variations in the composition and/or proportion of some of their components.

### *E. coli* O157:H7 Mainly Adheres to the ECM of Muscle Cells, But Similarly to the Different Myofibre Types

**FIGURE 2:**
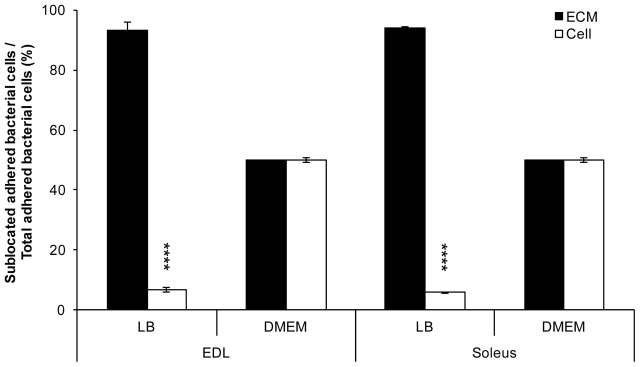
Proportion of *E. coli* O157:H7 adhering to EDL and soleus muscles as a function of their sublocation in the muscle tissue. Bacterial cells at the ECM and on the muscle cells were discriminated. The proportion of each of those bacteria first grown in LB or DMEM was calculated and based on bacterial cell count from epifluorescence microscopic observations on EDL or soleus cross-sections (**Figure 3**).

**FIGURE 3:**
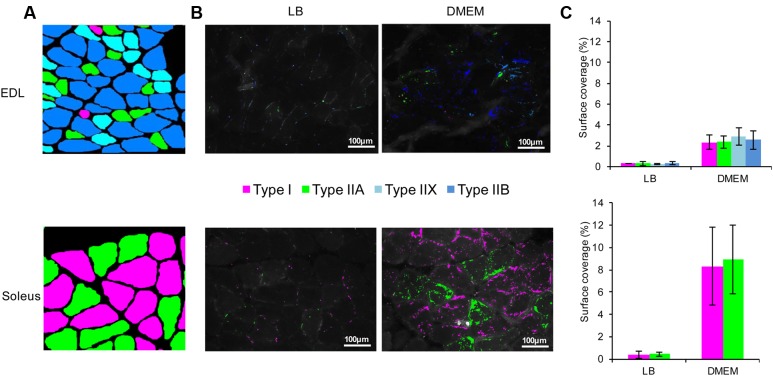
Adhesion of *E. coli* O157:H7 to the different types of myofibres constituent of the EDL and soleus muscles. **(A)** Each myofibre type was identified according to its MyHC isoform as described in the Materials and Methods. From all immunohistofluorescence images and for EDL and soleus, a virtual image was reconstructed showing the precise fibre type for each muscle cell. **(B)** On serial cross-sections of the EDL and soleus, the adhered *E. coli* O157:H7 CM454 pSaRe-Red1 first grown in LB or DMEM was visualised by epifluorescence microscopy. Fluorescent bacteria were stained virtually in colours corresponding to the myofibre type. **(C)** The level of adherent bacteria was assessed by the fluorescence intensity and expressed as the percentage of muscle cross-section surface covered by fluorescent bacterial cells.

Considering that the EDL and soleus essentially vary in the composition and proportion of myofibres of types I, IIA, IIX, and IIB, differential bacterial adhesion to these muscle cells was further investigated. For this purpose, serial cross-sections of EDL and soleus were performed. It first appeared whenever grown in LB or DMEM, *E. coli* O157:H7 CM454 adhered to muscle cells cross-sections, but unevenly since bacterial cells essentially localised at the muscle cells periphery (**Figures 2, 3**). With bacterial cells first grown in LB, more than 90% of adherent bacteria were essentially localised at the ECM of EDL and soleus muscle cells, respectively (**Figure 2**). In DMEM, half of the bacterial cells were found at the ECM (**Figure 2**) and the other half on the muscle fibres, especially on the edge of the cell (**Figure 3**).

Immunohistochemical analysis confirmed that, as a fast-twitch glycolytic skeletal muscle, the EDL contains the four main types of myofibres (I, IIA, IIX, IIX) but with the glycolytic fibres of types IIX and IIB in a much higher proportion, whereas, as an oxidative skeletal muscle, the soleus muscle was exclusively composed of oxidative fibres of types I and IIA (**Figure 3A**). Following myofibre typing, the cross-section muscle fibres present in EDL and soleus were further correlated with the adherent bacterial cells (**Figure 3B**). As expected from the results here above, it first appeared that the surface coverages of EDL or soleus muscle cross-sections was very significantly higher with *E. coli* O157:H7 CM454 grown in DMEM than in LB (**Figure 3C**); with respect to the different myofibre types and when grown in LB, bacterial surface coverages were about 0.3% on EDL and 0.4% on soleus, but reached 2.9 and 8.7% respectively when *E. coli* O157:H7 CM454 was grown in DMEM. However, we observed no difference in the bacterial surface coverages of type I, IIA, IIX, and IIB myofibres present in EDL or type I and IIA myofibres present in soleus (**Figures 3B,C**). Nonetheless, the surface coverage was still significantly higher for soleus than for EDL. This indicates that the different types of myofibres, with respect to their metabolic and/or contractile properties, did not influence the amount *E. coli* O157:H7 CM454 that adhered to meat. Altogether, differential *E. coli* O157:H7 tropism for oxidative over glycolytic skeletal muscle could not be explained by the differential proportion of contractile (types I vs. II) or metabolic (I and IIA vs. IIX and IIB) types of fibres.

### Postmortem Changes in Muscle Influences *E. coli* O157:H7 Adhesion to Muscle Fibres

**FIGURE 4:**
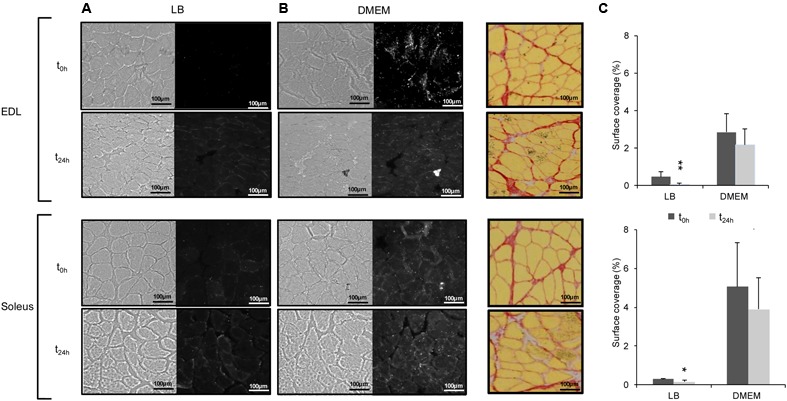
Adhesion of *E. coli* O157:H7 to EDL and soleus muscle cross-sections as a function of postmortem time. **(A)** Bacterial adhesion to EDL and soleus cross-sections was assessed at two postmortem times, *t*_0__h_ (i.e., 10 min after slaugther) and *t*_24__h_ (24 h after slaughter). For each muscle cross-section, left images show muscle fibres in bright field mode and right images show adhering fluorescent *E. coli* O157:H7 CM454 pSaRe-Red1. **(B)** Structural changes in the ECM (coloured in red) at *t*_0__h_ and *t*_24__h_ postmortem on serial cross-sections of EDL and soleus muscles. **(C)** The level of adherent bacteria was assessed by the fluorescence intensity and expressed as the percentage of muscle cross-section surface covered by fluorescent bacterial cells.

Considering that meat corresponds to the maturation of skeletal muscles after slaughtering, investigation of *E. coli* O157:H7 CM454 adhesion was further performed at two different postmortem times, namely t_0h_ and t_24h_. As previously observed above (**Figure 3**) and whatever the postmortem time, bacterial cells mainly co-localised at the ECM (**Figures 4A,B**). For *E. coli* O157:H7 CM454 first grown in LB, the surface coverages of EDL and soleus cross-sections were quite low and did not exceed 0.5 and 0.3%, respectively and were significantly lower at *t*_24__h_ than at *t*_0__h_ (**Figure 4C**). When grown in DMEM, however, the surface coverage was similar at both postmortem times but the surface coverage was again very significantly higher compared with *E. coli* O157:H7 CM454 grown in LB and higher for soleus cross-sections compared with EDL (**Figure 4C**); when *E. coli* O157:H7 CM454 was grown in DMEM, the bacterial surface coverage was about 2.5% on EDL at *t*_0__h_ and *t*_24h_, but reached 4.5% on soleus. Interestingly, both the EDL and soleus muscle tissues showed some structural changes at *t*_24__h_ postmortem, namely the size of intercellular spaces increased and the meshing of the ECM became loosen (**Figure 4B**).

## Discussion

Using the two well-characterised and extreme models of rat glycolytic and oxidative skeletal muscles, namely EDL and soleus, respectively, we show here that differential adhesion of *E. coli* O157:H7 occurred at the surface of skeletal muscles. The localisation trend observed at the surface of these whole skeletal muscles could be explained by variation in the organisation or thickness of the epimysium. Indeed, it is well known that the epimysium is a highly nonlinear ECM, which exhibits longitudinal periodicities (Gao et al., 2008; Gillies and Lieber, 2011). With respect to bovine muscle, and knowing that other skeletal muscles are in-between these two extremes, the white glycolytic EDL muscle (containing a high percentage of glycolytic myofibres but also some oxidative myofibres) is close to m. cutaneus trunci, biceps brachii, or latissimus dorsi, whereas the red oxidative soleus muscle (exclusively composed of oxidative myofibres) is close to the soleus and infraspinatus (Totland and Kryvi, 1991; Kirchofer et al., 2002). Rat EDL and soleus muscles are relevant models for simulating meat in lab experiments: (i) their muscular physiology is well-characterised, (ii) they show the same postmortem biochemical and structural changes as for farm animals, (iii) their maturation follows a faster evolution kinetics, which can be rigorously monitored and controlled over time (Chagnot et al., 2015a,b; Filgueras et al., 2016). In fact, these muscles can be extracted entirely without lesions, shortly after death of the animal and their size is compatible with microscopic observations, which makes them models of choice to investigate bacterial interactions at the tissue and cellular levels.

Besides the type of skeletal muscle, differential adhesion depended on anterior growth conditions. Growth of *E. coli* O157:H7 EDL933/CM454 in LB was previously shown to induce specific adhesion to some ECM fibrillar proteins, especially collagens I and III, whereas DMEM was clearly shown to induce self-aggregation, resulting in non-specific bacterial adhesion to ECM proteins (Chagnot et al., 2013a, 2014). Bacterial adhesion appeared to occur essentially around the muscles cells at the ECM and was significantly influenced by postmortem structural modifications. Recently, postmortem degradation of proteoglycans was suggested to destabilise the ECM and lead to the undocking of collagen fibres (Nishimura, 2010). Also, these postmortem structural modifications seems to affect the specific adhesion of bacteria to collagen as observed with bacterial cells grown in LB.

Whatever the different constituent myofibres of types I, IIA, IIX and IIB, however, no significant differences were observed for adhering bacteria. Muscle types, but not fibre types, affected the attachment of *E. coli* O157:H7, and this could be due to some variations in the ECM composition and/or organisation between the different muscles. The ECM at the periphery of the muscle fibres corresponds to the endomysium but the ECM at the surface of the muscle to the epimysium. Depending on the type of ECM at the different muscle structural organisation levels, the proportion of the different ECM proteins varies slightly (Gillies and Lieber, 2011; Hinds et al., 2011). From one muscle type to another, the composition of the ECM can also differ somewhat, as can the supramolecular organisation of the different components of the ECM, but elucidation is still needed.

By providing information on the spatial localisation of *E. coli* O157:H7 on meat, our results clearly demonstrate their ability to adhere to muscle tissue, especially at the ECM, which resulted in heterogeneous bacterial distribution in meat. *E. coli* O157:H7 exhibits a wide range of colonisation factors that could potentially participate in specific and non-specific adhesion to the ECM, e.g., several pili, flagella, and adhesins (Chagnot et al., 2012, 2013b; Monteiro et al., 2016). However, further studies are needed to determine the regulation and expression in different environmental conditions, as well as the exact contribution of each of these numerous molecular determinants of meat adhesion. For instance, the involvement of EhaB in binding to collagen or Cah in self-aggregation has been demonstrated, but the environmental conditions for their expression in *E. coli* O157:H7 have never been ascertained (Torres et al., 2002; Wells et al., 2009). In this regard, the present investigation opens the way to further characterisation of the molecular interactions between bacterial ECM-binding proteins and muscle ECM in different environmental conditions (Chagnot et al., 2012, 2013b).

This field of research has not attracted a lot of interest so far, but is promising and necessary. In the food industry and respective to HACCP (Hazard Analysis Critical Control Point), we could improve the preventive approach by designing measures to reduce the risks of ground beef meat contamination to an even safer level by considering differential *E. coli* O157:H7 adhesion and localisation to beef carcasses, with regards to different muscle types, bacterial behaviour in anterior and current environmental conditions, and heterogeneous bacterial distribution in meat with respect to ECM composition. Considering the meat ecosystem, understanding of the interactions of this foodborne pathogen with the food biotope and biocenosis, including the biotic and abiotic factors of the food matrix, could expedite development of novel preventive measures, such as the use of competitive microbial species for food biopreservation (Chaillou et al., 2015). Since the establishment of QMRA (quantitative microbial risk assessment) as the basis of food safety management, such information is required and is clearly highly relevant, but, as mentioned above, there are still major gaps in our basic knowledge. This also jeopardises the development of accurate predictive models incorporating the observations that food contamination with EHEC occurs at a low level, that the bacterial foodborne pathogen is physiologically diverse, and that the food matrix is heterogeneous. Besides its importance for risk assessment and for mitigation of EHEC contamination in the food chain and/or the food industry, knowledge of bacterial adhesion at molecular, cellular and tissue levels is also of great interest in the veterinary and/or medical sciences in relation to bacterial infection of muscle tissues.

## Author Contributions

CC, AV, TA, and MD conceived and designed the experiments. CC, AV, SR, and NC performed the experiments. CC, RT, TA, and MD analysed and interpreted the data. CC, AV, TA, and MD contributed reagents, materials, and analysis tools. CC, RT, TA, and MD wrote the paper.

## Conflict of Interest Statement

The authors declare that the research was conducted in the absence of any commercial or financial relationships that could be construed as a potential conflict of interest.
